# Imageless and image‐based robotic‐assisted total knee arthroplasty achieve equivalent radiographic accuracy: A randomised controlled trial

**DOI:** 10.1002/ksa.70123

**Published:** 2025-10-22

**Authors:** Rapeepat Narkbunnam, Yotsawadee Chorunchan, Keerati Chareancholvanich, Chaiwat Achawakulthep, Kit Awirotananon, Chaturong Pornrattanamaneewong

**Affiliations:** ^1^ Department of Orthopaedic Surgery, Faculty of Medicine Siriraj Hospital Mahidol University Bangkoknoi Bangkok Thailand

**Keywords:** imageless navigation, radiographic accuracy, robotic surgery, ROSA system, total knee arthroplasty

## Abstract

**Purpose:**

Robotic‐assisted total knee arthroplasty systems offer both image‐based and imageless workflows, but their comparative accuracy remains unclear. The robotic surgical assistant (ROSA) system uniquely provides both approaches within a single platform. This study compared radiographic accuracy between image‐based and imageless ROSA‐assisted total knee arthroplasty (ROSA‐TKA).

**Methods:**

This double‐blind, randomised controlled trial included patients with primary knee osteoarthritis randomised to imageless (*n* = 47) versus image‐based (*n* = 48) ROSA‐TKA. Primary outcomes assessed the differences between final intraoperative planned measurements (FIPM), robotic‐validated measurements (RVM) after bone cuts and postoperative scannogram measurements (PSM) across five radiographic alignment parameters measured at 12 weeks: hip‐knee‐ankle angle, femoral coronal and sagittal alignment, tibial coronal alignment and tibial slope. Secondary outcomes included functional scores and complications.

**Results:**

Both techniques demonstrated equivalent radiographic accuracy with no significant differences between groups. Mean differences between FIPM and RVM were ≤1 degree for all parameters, achieving 93.6%–100% accuracy within 2‐degree thresholds. No significant differences were observed between imageless and image‐based approaches in comparisons of FIPM and RVM with postoperative scanograms. However, these comparisons showed larger differences (1.2–2.5 degrees) and lower accuracy rates (48.9%–87.5% within 2‐degree thresholds), with sagittal alignment showing the greatest variation. Functional outcomes improved equally in both groups at 6 and 12 weeks, with comparable registration times and no complications.

**Conclusion:**

Both imageless and image‐based ROSA‐TKA achieved equivalent radiographic accuracy and clinical outcomes. The choice between techniques should be based on practical workflow considerations rather than accuracy concerns, as both approaches deliver comparable precision and excellent safety profiles.

**Clinical Trial Registration:**

This study was registered in the Thai Clinical Trials Registry (TCTR20240614001).

**Level of Evidence:**

Level I.

**Abbreviations**
2D: two‐dimensional
3D: three‐dimensional
CI: confidence intervals
CT: computed tomography
FCA: femoral coronal alignment
FIPM: final intraoperative planned measurements
FSA: femoral sagittal alignment
HKA: hip‐knee‐ankle angle
ICC: intraclass correlation coefficient
KOOS: knee injury and osteoarthritis outcome score
OKS: Oxford knee score
PACS: picture archiving and communication system
PSM: postoperative scannogram measurements
RCT: randomised controlled trial
ROSA: robotic surgical assistant
RVM: robotic‐validated measurements
r‐TKA: robotic‐assisted total knee arthroplasty
SNOSE: sequential numbered, opaque, sealed envelopes
SPSS: statistical package for the social sciences
TCA: tibial coronal alignment
TKA: total knee arthroplasty
TSA: tibial sagittal alignment

## INTRODUCTION

Robotic‐assisted total knee arthroplasty (r‐TKA) improves surgical precision and alignment reliability compared to conventional techniques [1, 2, 5, 6, 14, 15, 26, 28]. Two main robotic systems exist: image‐based and imageless. Image‐based systems use preoperative computer tomography (CT) scans or two‐dimensional (2D) radiographs to create patient‐specific models, providing detailed anatomy visualisation and accurate implant sizing but requiring additional radiation, cost and preparation time [34, 35]. Imageless systems perform intraoperative bone registration without preoperative imaging, offering reduced radiation, lower costs and streamlined workflow but depend on accurate registration and may have reduced precision in complex deformities [30].

The robotic surgical assistant (ROSA) knee system (Zimmer Biomet, 2019) uniquely offers both workflows within a single platform [16, 19, 24]. The image‐based version uses preoperative 2D radiographs converted to three‐dimensional (3D) images via X‐atlas Platform, while the imageless version performs intraoperative registration without preoperative imaging [10]. Literature demonstrates excellent precision for both approaches [33], with imageless ROSA achieving hip‐knee‐ankle angle (HKA) outlier rates of 0%–11% [4, 19, 20, 27, 28, 29, 32] and image‐based ROSA showing superior performance in planned alignment parameters [11]. Systematic reviews demonstrate that r‐TKA achieves higher surgical precision with significantly reduced radiological outliers compared to conventional total knee arthroplasty [14].

Despite growing evidence supporting both workflows, no randomised controlled trial (RCT) has directly compared image‐based versus imageless techniques within the same robotic platform. This RCT compared radiographic accuracy of implant positioning between image‐based and imageless ROSA‐assisted TKA, hypothesising that the image‐based approach would achieve enhanced reliability due to its preoperative planning capabilities and anatomical visualisation. Primary outcomes included HKA angle, femoral coronal (FCA) and sagittal alignment (FSA) and tibial coronal (TCA) and sagittal alignment (TSA). Determining equivalent performance of the imageless approach has significant implications for streamlining surgical workflows while ensuring reliability.

## MATERIALS AND METHODS

### Study design and patient population

**Figure 1:**
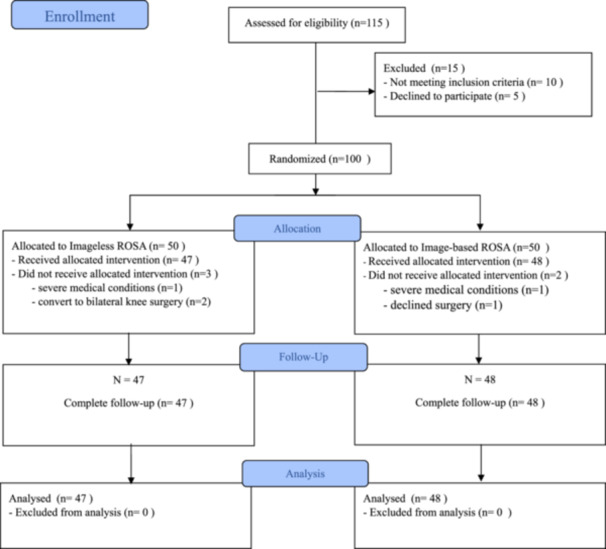
The CONSORT (consolidated standards of reporting trials) diagram.

This double‐blind, randomised controlled trial was conducted at a tertiary care hospital from July 2022 to February 2025. One hundred fifteen patients with primary knee osteoarthritis scheduled for unilateral TKA were assessed for eligibility. Fifteen patients were excluded (10 did not meet inclusion criteria, 5 declined participation), leaving 100 patients randomised into two equal groups (*n* = 50 each) comparing 2D radiographic‐based versus imageless ROSA‐TKA. All procedures were performed by two experienced arthroplasty surgeons using the ROSA robotic system with Persona TKA implants (Zimmer Biomet). Inclusion criteria included primary osteoarthritis meeting American College of Rheumatology criteria [3], unilateral TKA indication and age 18–80 years. Exclusion criteria included previous hip/knee/ankle surgery affecting limb alignment, varus/valgus deformity or flexion contracture exceeding 15 degrees, and conditions requiring revision implants. Five patients did not receive allocated intervention due to severe medical conditions (*n* = 2), conversion to bilateral TKA (*n* = 2) and patient declining surgery (*n* = 1). The remaining 95 patients (47 imageless ROSA, 48 image‐based ROSA) completed follow‐up and were included in final analysis. The CONSORT flow diagram is shown in Figure 1.

### Randomisation, allocation concealment and blinding

Randomisation was performed using computer‐generated sequences with mixed block sizes of 4. A central allocator, independent of patient recruitment and clinical care, generated the randomisation sequence and prepared sequential numbered, opaque, sealed envelopes (SNOSE) for allocation concealment. The sealed envelopes were opened by the scrub nurse immediately before surgery to ensure proper concealment until intervention. This double‐blind study maintained patient blinding throughout the study period, and outcome assessors measuring radiographic parameters were blinded to group allocation during data analysis. All patients underwent identical preoperative assessments including demographic data collection, functional score evaluation and standing full weight‐bearing scanograms according to the X‐atlas protocol regardless of group assignment. Radiographic measurements were performed using the picture archiving and communication system (PACS) (Synapse version 3.2.1). Postoperative care protocols, follow‐up schedules and outcome measurement procedures were standardised and identical for both treatment groups to eliminate potential bias in surgical outcome assessment.

### Surgical technique

At our institution, both conventional and r‐TKA procedures are performed. For this study, all eligible patients were offered participation in the robotic‐assisted protocol, with conventional TKA remaining available for patients who declined study participation or did not meet inclusion criteria.

All ROSA‐TKA procedures were performed under 300 mmHg tourniquet pressure using medial parapatellar approach with extra‐incisional bone array pin placement. The surgical workflow was identical for all patients, with bony landmark registration performed on femur and tibia according to ROSA protocol. In the image‐based ROSA group, surgeons used preoperative films converted into 3D images through X‐atlas Platform, allowing complete surgical planning including virtual knee modelling, implant sizing and predetermined cutting angles with verifiable bony registration points that could be re‐registered if inaccurate. In the imageless ROSA group, the 3D knee model was displayed but was not representative of actual patient anatomy, preventing verification of bony registration points.

All procedures followed functional alignment principles. After registration, systematic intraoperative soft tissue assessment was performed at extension (manual valgus‐varus stress testing at 10 degrees knee flexion) and flexion (distraction spoons at 90 degrees). The robotic system enabled precise component positioning while maintaining alignment parameters according to functional alignment protocol guidelines: HKA angle of 174–180 degrees; femoral component positioning in 3 degrees varus to 6 degrees valgus with 0–10 degrees flexion; femoral rotation of 0 degrees IR‐6 degrees ER (PCA) and 3 degrees IR‐6 degrees ER (TEA); tibial component parameters of 0–6 degrees varus with posterior slope of 0–5 degrees depending on implant type. The system prioritised preserving natural soft tissue balance with minimal releases when necessary, while maintaining these alignment boundaries to achieve balanced flexion‐extension gaps.

Based on intraoperative planning, the ROSA robotic arm executed the surgical plan with femoral distal resection, tibial proximal resection and femoral 4‐in‐1 positioning. Cut validation was performed, the 4‐in‐1 Persona cutting guide finished the distal femur, and cemented prostheses were implanted without patellar resurfacing.

### Outcome measurements

**Figure 2:**
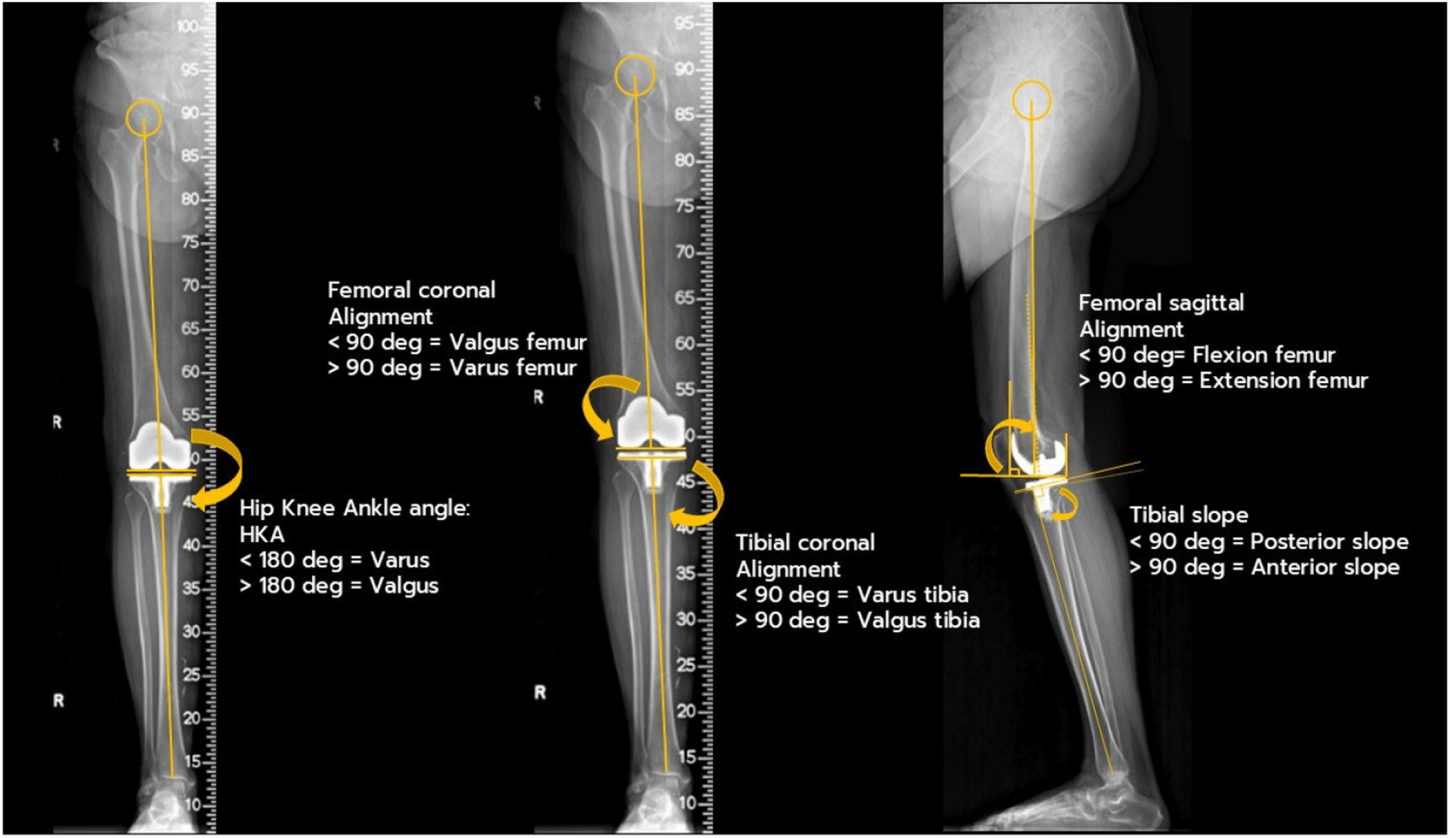
Measurement of postoperative scanogram in coronal and sagittal view.

The primary outcome was radiographic accuracy, measured as the absolute mean differences between final intraoperative planned measurements (FIPM), robotic‐validated measurements (RVM) after bone cuts and postoperative scannogram measurements (PSM) after TKA. Five key radiographic parameters were measured at 12 weeks postoperatively using standardised scannogram anteroposterior and lateral views (Figure 2): HKA angle representing overall limb alignment measured from femoral head centre to ankle centre through intercondylar notch, with values less than 180° indicating varus and greater than 180° indicating valgus alignment; femoral coronal alignment (FCA) measuring component deviation from the femoral mechanical axis; femoral sagittal alignment (FSA) assessing component flexion positioning relative to the distal femoral cortex; tibial coronal alignment (TCA) evaluating component angulation relative to the tibial mechanical axis; and tibial sagittal alignment (TSA) assessing posterior slope positioning relative to the tibial anatomical axis.

All radiographic measurements were performed using the PACS by two independent experienced arthroplasty surgeons who remained blinded to patient group assignments throughout the assessment process. Standardised patient positioning protocols included weight‐bearing anteroposterior scanograms with patella facing forward and lateral radiographs with 0–5 degrees knee flexion. The intraclass correlation coefficient (ICC) for interrater reliability ranged from 0.922 to 0.984, and intrarater reliability was assessed using repeated measurements on 20 randomly selected cases after 2 weeks, achieving ICC values of 0.915–0.978. All measurements were consistently reported to one decimal place accuracy to match the PACS measurement precision capabilities.

Secondary outcomes included intraoperative registration times for femoral and tibial bones measured to 0.1‐min precision, functional outcomes assessed through the knee injury and osteoarthritis outcome score (KOOS) [7] and Oxford knee score (OKS) [8] at 6 and 12 weeks, and comprehensive safety monitoring for intraoperative and early postoperative complications. Patient evaluations followed a structured timeline with baseline assessments including demographic data and initial functional scoring, intraoperative measurements and registration time recording, 6‐week clinical assessments with functional scoring, and 12‐week final radiographic measurements with repeated functional outcomes evaluation.

### Sample size calculation

Sample size was calculated using the difference in means with an effect size of 0.6, based on expected differences in radiographic accuracy of HKA between groups. With 80% power (*β* = 0.2) and 5% significance level (*α* = 0.05), using data from previous studies [16, 17, 19], the calculated sample size was 84 patients (42 per group). To account for a 10% dropout rate, the total of 100 patients were recruited in our study (50 knees per group).

### Enhanced Institutional Review Board (IRB) statement

This study received IRB approval from Siriraj Hospital, Mahidol University. Ethics Committee (approval number: COA514/2022) and was registered in the Thai Clinical Trials Registry (TCTR20240614001). Written informed consent was obtained from all participants prior to enrolment.

### Statistical analyses

Statistical analysis was performed using statistical package for the social sciences (SPSS) program version 18.0. Continuous variables were assessed for normal distribution using Shapiro–Wilk test. Normally distributed data were presented as mean ± standard deviation and compared using independent *t*‐test. Nonparametric data were presented as median with interquartile range and compared using Mann–Whitney *U* test. Categorical variables were presented as percentage and compared using chi‐square test or Fisher's exact test. For comparing differences among FIPM, RVM and PSM, analysis of variance (ANOVA) was used for normally distributed data and Kruskal–Wallis test for nonnormal distribution. Interrater reliability was assessed using ICC with 95% confidence intervals (CIs). The *p*‐value < 0.05 was set as the statistical significance.

## RESULTS

Patient characteristics were comparable between groups (Table 1), with mean ages of 69.5 ± 5.3 years (imageless ROSA) and 69.0 ± 7.1 years (image‐based ROSA). Both groups were predominantly women with Kellgren–Lawrence [13] grade 4 osteoarthritis and similar baseline HKA angles of 172.4 ± 8.5 degrees (imageless) and 173.5 ± 6.5 degrees (image‐based).

**Table 1:** Patients' characteristic of both groups.

Charateristic	Imageless ROSA (*n* = 47)	Image‐based ROSA (*n* = 48)
Age (year)	69.5 ± 5.3	69.0 ± 7.1
Body mass index (kg/m^2^)	26.6 ± 3.8	26.4 ± 4.7
Female gender (%)	38 (80.9%)	40 (83.3%)
Right side (%)	26 (55.3%)	29 (60.4%)
Kellgren–Lawrence grade		
Grade 3	3 (6.4%)	1 (2.1%)
Grade 4	44 (93.6%)	47 (97.9%)
Preoperative range of motion (deg)		
Flexion	110.1 ± 10.1	108.5 ± 10.1
Extension	1.5 ± 3.3	0.9 ± 3.2
Preoperative functional score		
OKS	25.2 ± 6.6	26.2 ± 7.2
KOOS	15.1 ± 5.1	14.0 ± 4.7
Preoperative radiographic parameters (deg)		
Hip‐knee‐ankle axis	172.4 ± 8.5	173.5 ± 6.5
Femoral coronal alignment	88.4 ± 2.3	88.4 ± 2.6
Femoral sagittal alignment	88.2 ± 2.4	88.8 ± 3.2
Tibial coronal alignment	91.2 ± 5.4	92.5 ± 3.8
Tibial sagittal alignment	80.0 ± 4.8	80.4 ± 4.7

Abbreviations: KOOS, knee injury and osteoarthritis outcome score; OKS, Oxford knee score; ROSA, robotic surgical assistant.

Radiographic accuracy analysis demonstrated excellent precision for both techniques. The absolute mean differences between FIPM and RVM were ≤1 degree across all five parameters in both groups, confirming superior robotic system accuracy. Comparisons involving postoperative scanogram measurements (FIPM vs. PSM and RVM vs. PSM) showed larger differences ranging 1.25–2.51 degrees, with overall statistical significance for FCA (*p* = 0.02) but no significant differences for other parameters (Table 2). Individual group comparisons revealed two isolated significant differences: imageless ROSA achieved better accuracy for FSA in FIPM versus RVM analysis (0.6 ± 0.6 vs. 0.8 ± 0.5 degrees, *p* = 0.033), while image‐based ROSA demonstrated superior accuracy for FCA in RVM versus PSM comparison (1.2 ± 1.0 vs. 1.7 ± 1.1 degrees, *p* = 0.032). Although statistical analysis revealed these isolated significant differences in femoral sagittal and coronal alignment, the actual magnitude remained well below 1 degree for FSA and within 1.7 degrees for FCA. These differences, while statistically significant, are unlikely to have clinical relevance given that they fall well within commonly accepted surgical tolerance thresholds (Table 3).

**Table 2:** The absolute mean differences of five radiographic parameters among the final intraoperative planned (FIPM), robotic‐validated (RVM) and postoperative scanogram measurements (PSM).

	FIPM vs. RVM		FIPM vs. PSM		RVM vs. PSM		*p* value
	Mean ± SD	95% confidence interval (CI)	Mean ± SD	95% CI	Mean ± SD	95% CI	
Hip‐knee‐ankle angle							0.661
Imageless	0.51 ± 0.86	0.26–0.76	2.01 ± 1.42	1.59–2.42	2.02 ± 1.35	1.62–2.41	
Image‐based	0.38 ± 0.69	0.18–0.58	1.66 ± 1.29	1.28–2.03	1.79 ± 1.23	1.43–2.15	
Femoral coronal alignment							0.020
Imageless	0.35 ± 0.40	0.23–0.46	1.62 ± 1.18	1.27–1.96	1.65 ± 1.08	1.34–1.97	
Image‐based	0.45 ± 0.51	0.30–0.60	1.25 ± 1.24	0.89–1.60	1.19 ± 0.99	0.90–1.48	
Femoral sagittal alignment							0.349
Imageless	0.57 ± 0.58	0.40–0.74	2.49 ± 1.64	2.01–2.97	2.51 ± 1.75	1.99–3.02	
Image‐based	0.82 ± 0.54	0.67–0.97	2.42 ± 1.67	1.93–2.90	2.30 ± 1.80	1.78–2.82	
Tibial coronal alignment							0.347
Imageless	0.46 ± 0.70	0.26–0.67	1.70 ± 1.52	1.25–2.14	1.78 ± 1.63	1.30–2.26	
Image‐based	0.47 ± 0.56	0.29–0.65	1.33 ± 1.47	0.90–1.75	1.54 ± 1.50	1.11–1.98	
Tibial sagittal alignment							0.948
Imageless	0.61 ± 0.72	0.39–0.82	2.14 ± 1.41	1.72–2.55	2.17 ± 1.39	1.76–2.58	
Image‐based	0.44 ± 0.56	0.29–0.61	2.02 ± 1.36	1.62–2.42	2.09 ± 1.43	1.68–2.51	

**Table 3:** Individual comparison of measurement accuracy between FIPM, RVM and PSM measurements in imageless versus image‐based ROSA.

Parameters	Imageless ROSA (*n* = 47)	Image‐based ROSA (*n* = 48)	Mean diff (95% confidence interval)	*p* value
FIPM vs. RVM (deg)				
HKA	0.5 ± 0.9	0.4 ± 0.7	0.1 (‐0.2, 0. 5)	0.406
FCA	0.3 ± 0.4	0.4 ± 0.5	‐0.1 (‐0.3, 0.1)	0.286
FSA	0.6 ± 0.6	0.8 ± 0.5	−0.3 (−0.5, 0)	0.033
TCA	0.5 ± 0.7	0.5 ± 0.6	0 (−0.3, 0.3)	0.971
TSA	0.6 ± 0.7	0.4 ± 0.6	0.2 (−0.1, 0.4)	0.234
FIPM vs. PSM (deg)				
HKA	2.0 ± 1.4	1.7 ± 1.3	0.3 (−0.2, 0.9)	0.208
FCA	1.6 ± 1.2	1.2 ± 1.2	0.4 (−0.1, 0.9)	0.138
FSA	2.5 ± 1.6	2.4 ± 1.7	0.1 (−0.6, 0.7)	0.831
TCA	1.7 ± 1.5	1.3 ± 1.5	0.4 (−0.2, 1.0)	0.227
TSA	2.1 ± 1.4	2.0 ± 1.4	0.1 (−0.4, 0.7)	0.681
RVM vs. PSM (deg)				
HKA	2.0 ± 1.4	1.8 ± 1.2	0.2 (−0.3, 0.8)	0.390
FCA	1.7 ± 1.1	1.2 ± 1.0	0.5 (0, 0.9)	0.032
FSA	2.5 ± 1.8	2.3 ± 1.8	0.2 (−0.5, 0.9)	0.568
TCA	1.8 ± 1.6	1.5 ± 1.5	0.3 (−0.4, 0.9)	0.468
TSA	2.2 ± 1.4	2.1 ± 1.4	0.1 (−0.5, 0.7)	0.793

Abbreviations: FCA, femoral coronal alignment; FIPM; final intraoperative planned; FSA, femoral sagittal alignment; HKA, hip‐knee‐ankle angle; PSM, postoperative scanogram; ROSA, robotic surgical assistant; RVM, robotic‐validated; TCA, tibial coronal alignment; TSA, tibial sagittal alignment.

**Figure 3:**
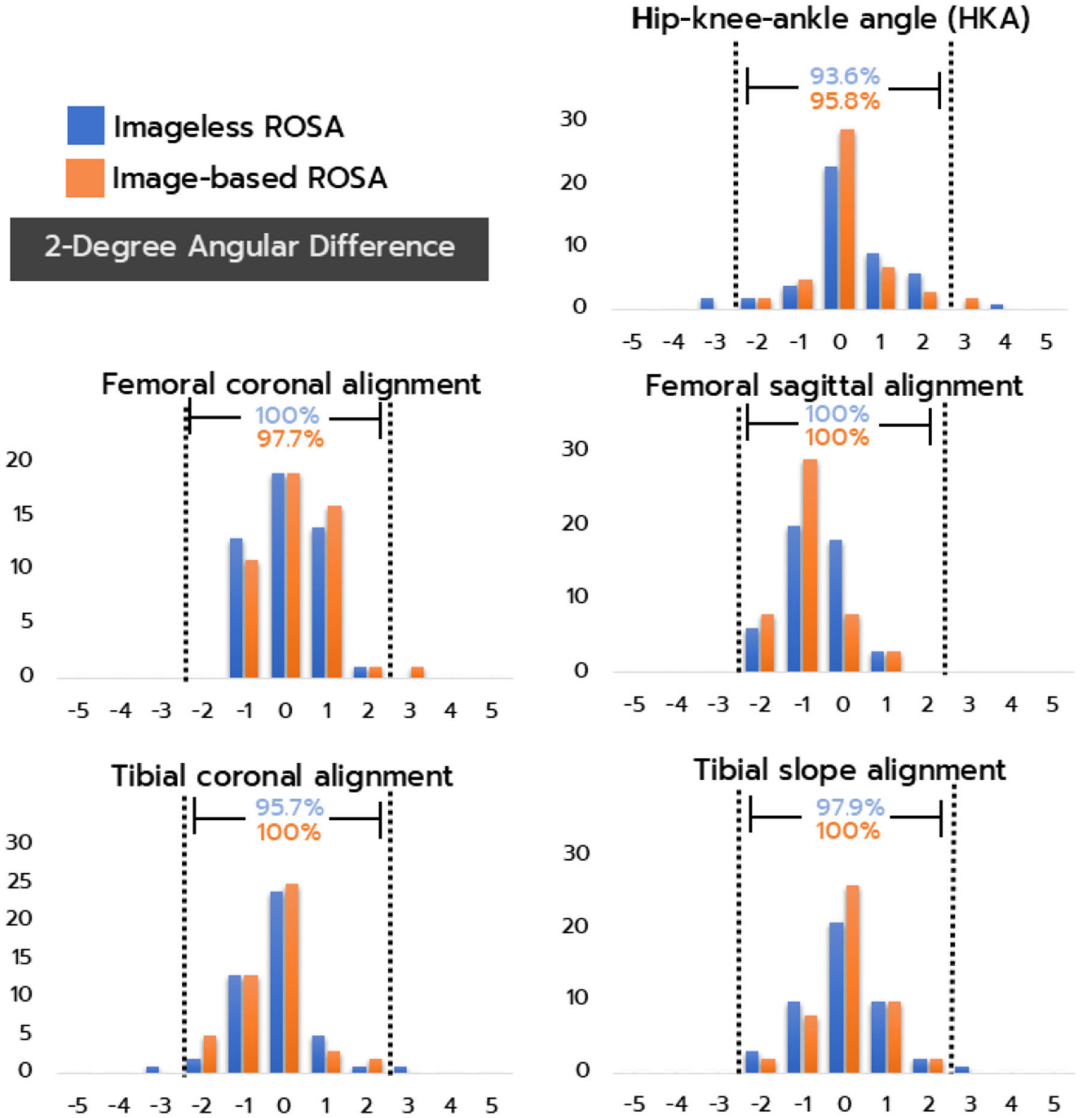
Final intraoperative planned measurements versus robotic‐validated measurements alignment distribution with 2‐degree angular difference analysis in robotic surgical assistant (ROSA)‐assisted total knee arthroplasty.

**Figure 4:**
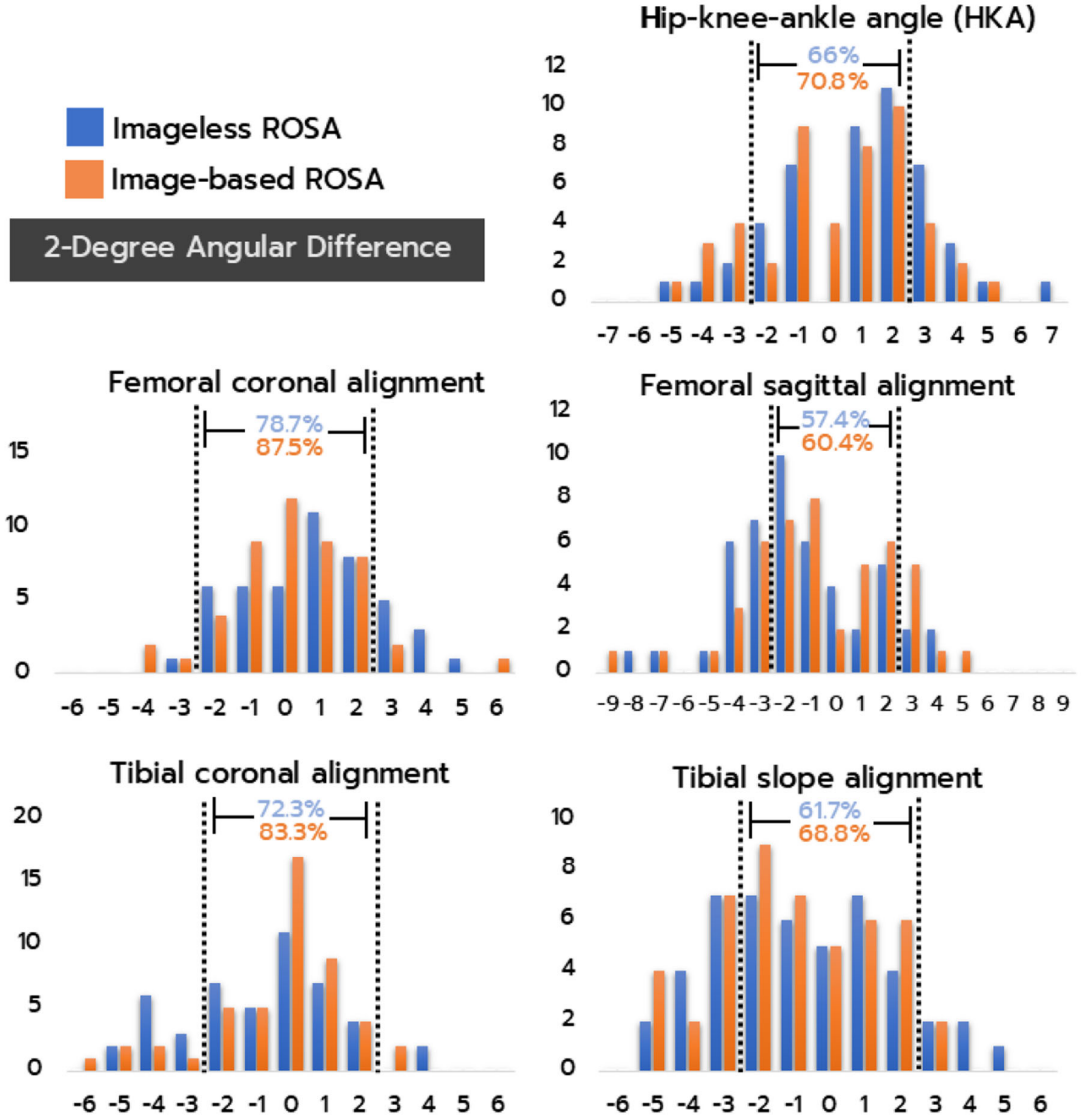
Final intraoperative planned measurements versus postoperative scannogram measurements alignment distribution with 2‐degree angular difference analysis in robotic surgical assistant (ROSA)‐assisted total knee arthroplasty.

**Figure 5:**
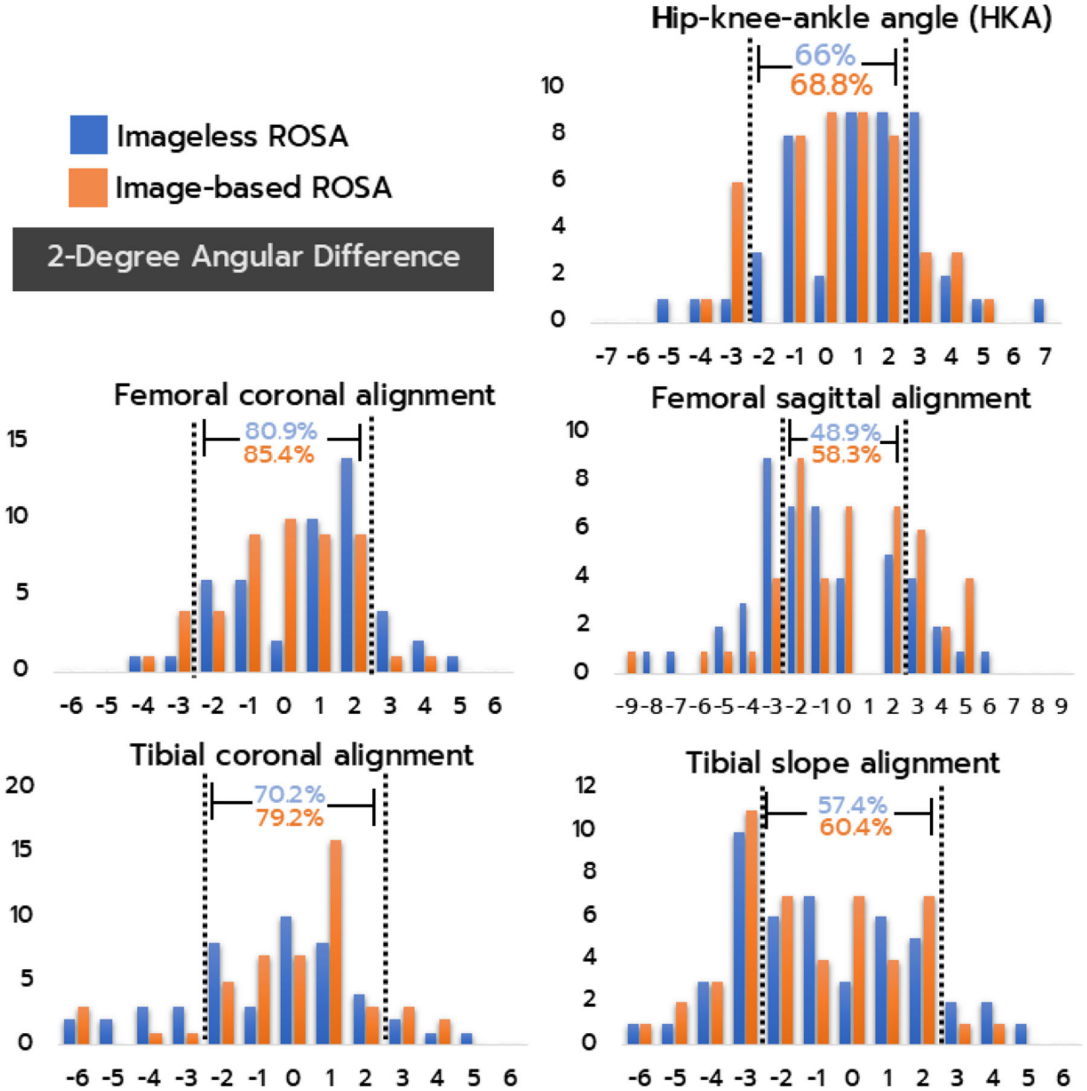
Robotic‐validated measurements versus postoperative scannogram measurements alignment distribution with 2‐degree angular difference analysis in robotic surgical assistant (ROSA)‐assisted total knee arthroplasty.

Angular threshold analysis demonstrated equivalent accuracy between techniques at 2‐ and 3‐degree cutoffs (all *p* > 0.05). FIPM versus RVM comparisons achieved excellent accuracy with 93.6%–100% of measurements within 2‐degree thresholds. Comparisons involving scanogram measurements showed lower accuracy rates, particularly for sagittal parameters, with FSA demonstrating the poorest performance at 48.9%–58.3% accuracy within 2‐degree thresholds for RVM versus PSM comparisons (Table 4, Figures 3, 4, 5). Measurement reliability was excellent with ICC values ranging from 0.922 to 0.984.

**Table 4:** Angular difference analysis comparing imageless and image‐based ROSA at 2‐ and 3‐degree thresholds.

Parameters	Difference within 2 degrees			Difference within 3 degrees		
	Imageless ROSA (*n* = 47)	Image‐based ROSA (n = 48)	*p*‐Value	Imageless ROSA (*n* = 47)	Image‐based ROSA (*n* = 48)	*p* value
FIPM vs. RVM (deg)						
HKA	93.6%	95.8%	0.677	97.9%	100%	0.495
FCA	100%	97.9%	1.000	100%	100%	‐
FSA	100%	100%	‐	100%	100%	‐
TCA	95.7%	100%	0.242	100%	100%	‐
TSA	97.9%	100%	0.495	100%	100%	‐
FIPM vs. PSM (deg)						
HKA	66.0%	70.8%	0.609	87.2%	89.6%	0.720
FCA	78.7%	87.5%	0.253	91.5%	93.8%	0.714
FSA	57.4%	60.4%	0.769	76.6%	83.3%	0.412
TCA	72.3%	83.3%	0.197	78.7%	89.6%	0.147
TSA	61.7%	68.8%	0.471	80.9%	87.5%	0.374
RVM vs. PSM (deg)						
HKA	66.0%	68.8%	0.772	85.1%	85.4%	0.966
FCA	80.9%	85.4%	0.552	91.5%	95.8%	0.435
FSA	48.9%	58.3%	0.358	76.6%	79.2%	0.763
TCA	70.2%	79.2%	0.315	80.9%	87.5%	0.374
TSA	57.4%	60.4%	0.769	83.0%	85.4%	0.745

Abbreviations: FCA, femoral coronal alignment; FIPM; final intraoperative planned; FSA, femoral sagittal alignment; HKA, hip‐knee‐ankle angle; PSM, postoperative scanogram; ROSA, robotic surgical assistant; RVM, robotic‐validated; TCA, tibial coronal alignment; TSA, tibial sagittal alignment.

Secondary outcomes revealed comparable registration times between groups and no significant differences in KOOS and OKS scores at 6 and 12 weeks (Table 5). No complications including venous thromboembolism, surgical site infection, or periprosthetic fracture occurred in either group.

**Table 5:** Functional scores and perioperative outcomes between imageless and image‐based robotic surgical assistant (ROSA) groups.

Outcomes	Imageless ROSA (*n* = 47)	Image‐based ROSA (*n* = 48)	*p* value
Intraoperative outcomes			
Femoral registration time (min)	2.1 (1.4–2.4)	1.6 (1.4–2.3)	0.170
Tibial registration time (min)	0.5 (0.4–1.0)	0.4 (0.3–1.1)	0.660
Postoperative outcomes			
Oxford knee score (OKS) at 6 weeks	29.1 ± 8.2	26.7 ± 7.8	0.152
Knee injury and osteoarthritis outcome score (KOOS) at 6 weeks	14.3 ± 6.4	14.7 ± 4.8	0.789
OKS at 12 weeks	33.8 ± 8.4	33.7 ± 7.8	0.929
KOOS at 12 weeks	9.6 ± 4.8	11.0 ± 4.8	0.173

## DISCUSSION

This randomised controlled trial represents the first head‐to‐head comparison of imageless versus image‐based ROSA‐assisted TKA. Our results demonstrated that both techniques achieved equivalent radiographic accuracy with predominantly no significant differences in alignment parameters, functional outcomes, or safety profiles. These findings challenge the prevailing assumption that preoperative image‐based planning necessarily provides superior accuracy compared to imageless registration.

Our FIPM versus RVM analysis demonstrated mean differences ≤1 degree for all parameters in both groups, consistent with previous studies by Hasegawa et al. [11], Rossi et al. [25] and Vanlommel et al. [32] showing excellent robotic accuracy. Recent systematic reviews corroborated these findings [2, 9, 33]. Although statistical analysis revealed isolated significant differences in FCA (*p* = 0.02), the actual magnitude remained within 1 degree, confirming inherent robotic system accuracy for both techniques. Contemporary studies using the ROSA system have similarly demonstrated superior precision in restoring joint line height and posterior condylar offset compared to conventional manual TKA [23], with mean changes of 0.2 mm for joint line height and 0.02 mm for posterior condylar offset [18].

When comparing robotic values (FIPM and RVM) with PSM, our study found differences ranging 1.2–2.5 degrees, with sagittal parameters consistently showing greater variations than coronal parameters. Although individual analysis revealed two statistically significant differences—imageless ROSA for FSA in FIPM versus RVM comparisons (0.6 ± 0.6 vs. 0.8 ± 0.5 degrees, *p* = 0.033) and image‐based ROSA for FCA in RVM versus PSM comparisons (1.2 ± 1.0 vs. 1.7 ± 1.1 degrees, *p* = 0.032)—the actual magnitude of these differences remained well below 1 degree, suggesting limited clinical significance. These findings were consistent with Shin et al. [29], who reported overall differences of 0.88–2.04 degrees between robotic and radiographic measurements, with sagittal values reaching approximately 2 degrees. However, contrasting evidence suggested deviations of less than 1 degree when using navigated measurement systems rather than scanograms [19]. The measurement errors observed in scanograms may be attributed to several factors, including limb rotation effects and inherent limitations in sagittal plane assessment. Studies have demonstrated that rotation from 20 degrees external to 20 degrees internal can alter FCA by 4 degrees and TCA by 2 degrees [17, 22]. Furthermore, sagittal plane measurements on scanograms are particularly susceptible to patient positioning errors and beam angulation variations, which may explain the consistently lower accuracy rates observed for FSA and TSA parameters compared to coronal measurements. Despite these potential sources of error, scanograms remained more practical than CT scans in our healthcare setting due to cost considerations and reduced waiting times, and we implemented standardised postoperative radiographic protocols to minimise rotational errors.

When analysing angular differences at 2‐degree thresholds, FIPM versus RVM comparisons achieved excellent accuracy with 93.6%–100% of measurements within acceptable limits for both techniques, demonstrating superior robotic precision. However, comparisons involving PSM showed considerably lower accuracy rates, ranging from 48.9% to 87.5% for FIPM versus PSM and 48.9%–85.4% for RVM versus PSM, with sagittal parameters (FSA and TSA) consistently showing the lowest accuracy rates. Notably, FSA demonstrated the poorest performance with only 48.9% (imageless) and 58.3% (image‐based) of measurements within 2‐degree thresholds for RVM versus PSM comparisons. These variations might be explained by inherent mechanical limitations of the robotic cutting system, where the robotic arm attachment functions as a cutting guide with a standardised slot thickness of 1.27 mm to accommodate the saw blade. This design might introduce toggle or slight movement of the saw blade within the slot, translating into small angular errors during bone preparation. Previous biomechanical studies support this hypothesis, demonstrating that TKA bone cuts performed with 1.19 mm blades resulted in errors of 1.3 degrees in the sagittal plane and 0.4–0.8 degrees in the coronal plane [21]. However, using the clinically accepted 3‐degree threshold for conventional TKA, both techniques demonstrated excellent safety boundaries for all parameters, with HKA achieving 85.1%–100% accuracy rates and most other parameters exceeding 80%–95%. These results were similar to previous studies [33].

The comparable accuracy between two techniques suggested that the option should be based on practical considerations rather than precision concerns. The clinical significance of these findings extends beyond mere accuracy comparisons. The similar performance of imageless ROSA‐TKA suggests that surgeons can achieve precise implant positioning without the additional radiation exposure, cost and workflow complexity associated with preoperative imaging. This has important implications for healthcare resource allocation, patient convenience and surgical efficiency, particularly in high‐volume arthroplasty centres.

Image‐based ROSA‐TKA offered preoperative visualisation and planning capabilities, allowing surgeons to assess bone anatomy, implant sizing and potential surgical challenges before surgery. However, this advantage came with additional workflow requirements, including preoperative scanograms acquisition and 7–14 days of processing time. Imageless ROSA‐TKA provided immediate workflow flexibility without preoperative imaging requirements, potentially reducing healthcare costs and patient visits. Our registration times of 2–3 min for femoral and tibial bones suggested minimal operative time impact for both techniques. Therefore, the technique selection might depend on surgeon preferences, institutional resources and case complexity rather than accuracy concerns.

Beyond these considerations, both ROSA techniques demonstrated unique advantages in the context of personalised alignment strategies. The functional alignment approach we employed prioritised soft tissue balance and individualised component positioning within defined parameters rather than targeting neutral mechanical axis [12, 31]. Image‐based ROSA provided valuable preoperative visualisation for planning component positioning within functional alignment boundaries, particularly for assessing individual anatomical variations that influence rotational alignment targets. However, imageless ROSA's real‐time intraoperative flexibility proved equally effective for functional alignment execution, as this philosophy relies heavily on dynamic soft tissue assessment and gap balancing during surgery. Our findings suggest that both robotic approaches can successfully implement functional alignment principles, with the choice potentially depending more on institutional considerations than on alignment philosophy requirements. Future research should investigate how these robotic approaches perform across different personalised alignment strategies including kinematic and restricted kinematic alignment.

For the secondary outcomes, the similar functional outcomes observed at 6 and 12 weeks supported our primary findings. No complications occurred in our study, confirming the safety of both techniques. While these short‐term results are encouraging, longer‐term follow‐up is needed to establish the relationship between radiographic accuracy and implant longevity in robotic TKA.

### Limitations

Several limitations warrant consideration. First, all ROSA‐TKA procedures were performed by experienced surgeons, and results may differ in low‐volume hospitals or with less experienced operators. Since image‐based ROSA‐TKA provides virtual knee models representing actual patient anatomy, bony registration points can be verified to improve registration accuracy; therefore, we recommend beginner ROSA‐TKA surgeons use the image‐based technique during their learning curve. Second, our radiographic evaluation was limited to standard scanograms which cannot adequately assess rotational alignment of both femoral and tibial components. While intraoperative rotational measurements were recorded using PCA and TEA landmarks for femoral components and tibial tubercle‐PCL insertion relationship for tibial components, postoperative rotational accuracy assessment would require CT imaging or specialised radiographic techniques, representing an important limitation given the clinical significance of rotational malalignment in TKA outcomes. Third, the 12‐week follow‐up was appropriate for radiographic assessment but insufficient for long‐term functional outcomes, patient satisfaction and implant survival, requiring future studies with extended follow‐up periods. Fourth, as a single‐centre study performed by experienced surgeons, our results may not be directly generalisable to all clinical settings, particularly those with less robotic surgery experience or different institutional workflows.

## CONCLUSIONS

Both imageless and image‐based ROSA‐assisted TKA achieve equivalent radiographic accuracy and clinical outcomes, with minimal clinically insignificant differences between techniques. The choice of technique should be based on practical considerations including workflow preferences, cost considerations and surgeon experience rather than accuracy concerns. Both approaches deliver excellent clinical results with equivalent safety profiles, allowing surgeons to select based on institutional resources and individual patient needs.

## AUTHOR CONTRIBUTIONS

**Rapeepat Narkbunnam**: Conceptualisation; methodology; investigation; data collection; formal analysis; writing—original draft; writing—review and editing. **Yotsawadee Chorunchan**: Investigation; data collection; validation; writing—review and editing. **Keerati Chareancholvanich**: Conceptualisation; methodology; supervision; writing—review and editing. **Chaiwat Achawakulthep**: Investigation; data collection; validation; writing—review and editing. **Kit Awirotananon**: Investigation; data collection; validation; writing—review and editing. **Chaturong Pornrattanamaneewong**: Conceptualisation; methodology; supervision; project administration; writing—review and editing; corresponding author responsibilities.

## CONFLICT OF INTEREST STATEMENT

The authors declare no conflict of interest.

## ETHICS STATEMENT

This study was approved by Siriraj Institutional Review Board (COA 514/2022). All procedures performed in studies involving human participants were in accordance with the ethical standards of the institutional and/or national research committee and with the 1964 Helsinki declaration and its later amendments or comparable ethical standards. Written informed consent was obtained from all individual participants included in the study at the outpatient clinic.

## Supporting information

Supporting information.

## Data Availability

The data that support the findings of this study are available on request from the corresponding author. The data are not publicly available due to privacy or ethical restrictions.
