# Supplementary material for: Imageless and image‐based robotic‐assisted total knee arthroplasty achieve equivalent radiographic accuracy: A randomised controlled trial
Source: Knee Surg Sports Traumatol Arthrosc. 2025 Oct 22;34(6):2167–79. doi: 10.1002/ksa.70123 (PMC13266914; doi:10.1002/ksa.70123)
Supplement: Supplementary file 1 — Supporting information. [file KSA-34-2167-s001.docx]

Supplement Table 1 Raw Radiographic Measurements of Alignment Parameters for FIPM, RVM, and PSM in Imageless versus Image-based ROSA Groups

| Parameters | HKA(deg) | FCA(deg) | FSA(deg) | TCA(deg) | TSA (deg) | p-value |
| --- | --- | --- | --- | --- | --- | --- |
| FIPM |  |  |  |  |  |  |
| Imageless ROSA  (n = 47) | 178.55 ± 1.76 | 89.60 ± 1.55 | 87.09 ± 0.28 | 89.03 ± 1.11 | 85.30 ± 1.11 | 1.000 |
| Image-based ROSA  (n =48) | 179.34 ± 1.39 | 89.80 ± 0.70 | 87.00 ± 0.36 | 89.68 ± 0.81 | 85.33 ± 0.85 |  |
| RVM |  |  |  |  |  |  |
| Imageless ROSA  (n = 47) | 178.46 ± 2.04 | 89.52 ± 1.66 | 87.55 ± 0.70 | 89.21 ± 1.43 | 85.23 ± 1.46 | 1.000 |
| Image-based ROSA  (n =48) | 179.19 ± 1.56 | 89.59 ± 0.87 | 87.72 ± 0.71 | 89.94 ± 1.12 | 85.26 ± 1.09 |  |
| PSM |  |  |  |  |  |  |
| Imageless ROSA  (n = 47) | 177.57 ± 2.84 | 88.79 ± 2.26 | 88.47 ± 2.67 | 89.83 ± 2.16 | 86.00 ± 2.82 | 1.000 |
| Image-based ROSA  (n =48) | 178.94 ± 2.30 | 89.65 ± 1.70 | 87.71 ± 2.87 | 90.08 ± 1.62 | 86.38 ± 2.43 |  |
